# Effect of excitation power on voltage induced local magnetization dynamics in an ultrathin CoFeB film

**DOI:** 10.1038/s41598-017-02427-3

**Published:** 2017-05-24

**Authors:** Bivas Rana, Yasuhiro Fukuma, Katsuya Miura, Hiromasa Takahashi, YoshiChika Otani

**Affiliations:** 1 Center for Emergent Matter Science, RIKEN, 2-1 Hirosawa, Wako, 351-0198 Japan; 2 Department of Computer Science and Electronics, Kyushu Institute of Technology, 680-4 Kawazu, Iizuka, 820-8502 Japan; 3 Research and Development Group, Hitachi, Ltd., 1-280 Higashi-koigakubo, Kokubunji-shi Tokyo, 185-8601 Japan; 4 Institute for Solid State Physics, University of Tokyo, Kashiwa, 277-858 Japan

## Abstract

Voltage or electric field induced magnetization dynamics promises low power spintronics devices. For successful operation of some spintronics devices such as magnetic oscillators and magnetization switching devices a clear understanding of nonlinear magnetization dynamics is required. Here, we report a detailed experimental and micromagnetic simulation study about the effect of excitation power on voltage induced local magnetization dynamics in an ultrathin CoFeB film. Experimental results show that the resonance line-width and frequency remains constant, whereas cone angle of the magnetization precession increases linearly with square-root of excitation power below threshold value, known as linear excitation regime. Above threshold power, the dynamics enters into nonlinear regime where resonance line-width monotonically increases and resonance frequency monotonically decreases with increasing excitation power. Simulation results reveal that a strong nonlinear and incoherent magnetization dynamics are observed in our experiment above the threshold power which reduces dynamic magnetic signal by suppressing large cone angle of magnetization precession. Moreover, a significant transfer of spin angular momentum from uniform FMR mode to its degenerate spin waves outside of excitation area further restrict the cone angle of precession within only few degrees in our device. Our results will be very useful to develop all-voltage-controlled spintronics devices.

## Introduction

The recent discovery of voltage-controlled magnetic anisotropy (VCMA) has a huge potential for the development of low power spintronics devices fully operated by voltage^1, 2^. Several physical phenomena like control of coercive field^3^ and domain wall motion^4, 5^, magnetization switching^1, 6–9^ and coherent ferromagnetic resonance induced by voltage^10–12^ have already been studied by several groups. Although various kinds of material systems have been used for these studies, the multilayer systems formed by 3d-ferromagnetic metals and insulating MgO are found to be one of the most promising candidates among them. In particular, magnetic tunnel junctions (MTJ) formed by CoFeB/MgO/CoFeB are the basic structure of modern spintronics devices due to their large tunneling magnetoresistance (TMR) value at room temperature^13, 14^, which can be used in non-volatile MRAM devices.

Precessional magnetization dynamics are called in the linear regime when cone angle of magnetization precession remains only within few degrees. When the excitation power crosses threshold value, the trajectory of the magnetization precession is strongly modified and dominated by incoherent magnetization precession, known as nonlinear magnetization dynamics^15, 16^. The nonlinear magnetization dynamics are essential for the operation of spintronics devices such as magnetic switching devices^6, 7, 9^, magnetic oscillators^17^. Until now, most of the voltage induced ferromagnetic resonances are reported in the linear regime^10–12, 18^. In spite of many reports on the magnetic field^16, 19, 20^ or spin torque^15^ induced nonlinear magnetization dynamic, only a single report on voltage induced nonlinear dynamics of a spatially confined CoFeB film with perpendicular magnetic anisotropy (PMA) are found^21^. Here, we study VCMA induced nonlinear magnetization dynamics in an ultrathin CoFeB film. We locally excite magnetization dynamics and systematically increase excitation power to investigate the variation of resonance frequency, line-width, line-shape and cone angle of precession with excitation power. We also performed micromagnetic simulations to visualize the spatial maps of dynamic magnetization at different excitation power and to understand the underlying mechanism of observed dynamic behavior as a function of excitation power. We believe that our study will be very useful to understand the nonlinear magnetization dynamics in an ultrathin ferromagnetic film under local excitation scheme.

## Results

### Schematic of device and working principle

**Figure 1:**
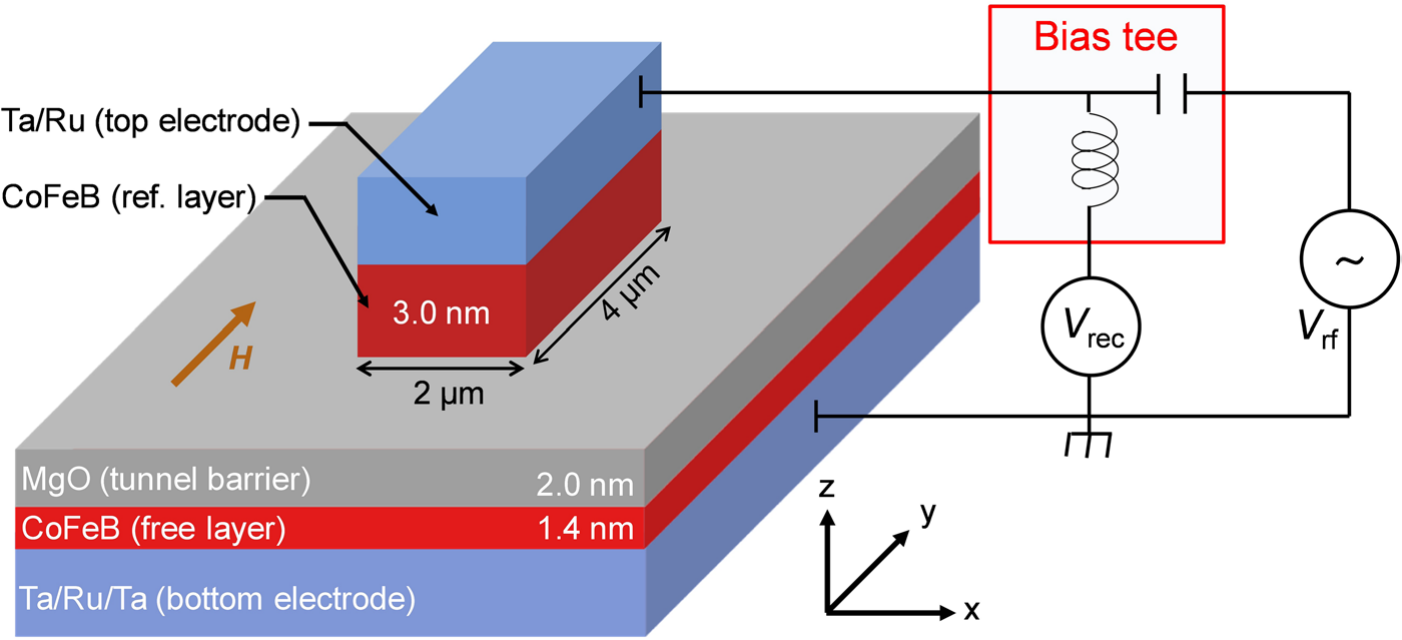
Schematic diagram of device and experimental set up. RF signals are sent through capacitor port of bias tee and rectified voltages are measured through dc port of bias tee.

Figure 1 shows the schematic diagram of the device and experimental set up. The device with MTJ structure consists of 3 nm top CoFeB layer (reference layer) and 1.4 nm bottom CoFeB layer (free layer) separated by a 2 nm thick insulating MgO layer (see methods for details). The reference layer was patterned into rectangular shape (2 × 4 µm^2^) in the middle of a much wider (50 × 100 µm^2^) free layer (Fig. 1). Both CoFeB layers have PMA at the interface of CoFeB/MgO due to the hybridization of Fe-3d and O-2p orbitals^22, 23^ and the magnitude of PMA strongly depends upon the thickness of ferromagnetic layer (inversely proportional to thickness)^24, 25^. The PMA field value of reference layer is less than the demagnetizing field. Therefore, reference layer has in-plane magnetic easy axis^24^. On the other hand, the PMA of free layer overcomes the demagnetizing field to have a net effective PMA. Therefore, free layer has out-of-plane magnetic easy axis. When dc or rf voltage is applied across the MTJ, the electric field at the interface of CoFeB layers modulates charge or spin density in the Fe-3d orbitals^26–28^, which causes a change in PMA. This change in PMA can be thought as equivalent to a magnetic field along out-of-plane direction which is proportional to out-of-plane component of saturation magnetization (*M*_z_)^10^. For our measurement, rf voltage (*V*_rf_) with varying frequency (*f*) is applied across the MTJ through the capacitor port of bias tee (Fig. 1). When *f* matches with the ferromagnetic resonance frequency (*f*_FMR_) of the free layer under the application of bias magnetic field (*H*), its magnetization oscillates around effective field direction. The rectified voltage (*V*_rec_) is measured through the dc port of bias tee as a function of *f* varying from 2 to 9 GHz (see methods for details).

### Variation of PMA field with dc voltage

**Figure 2:**
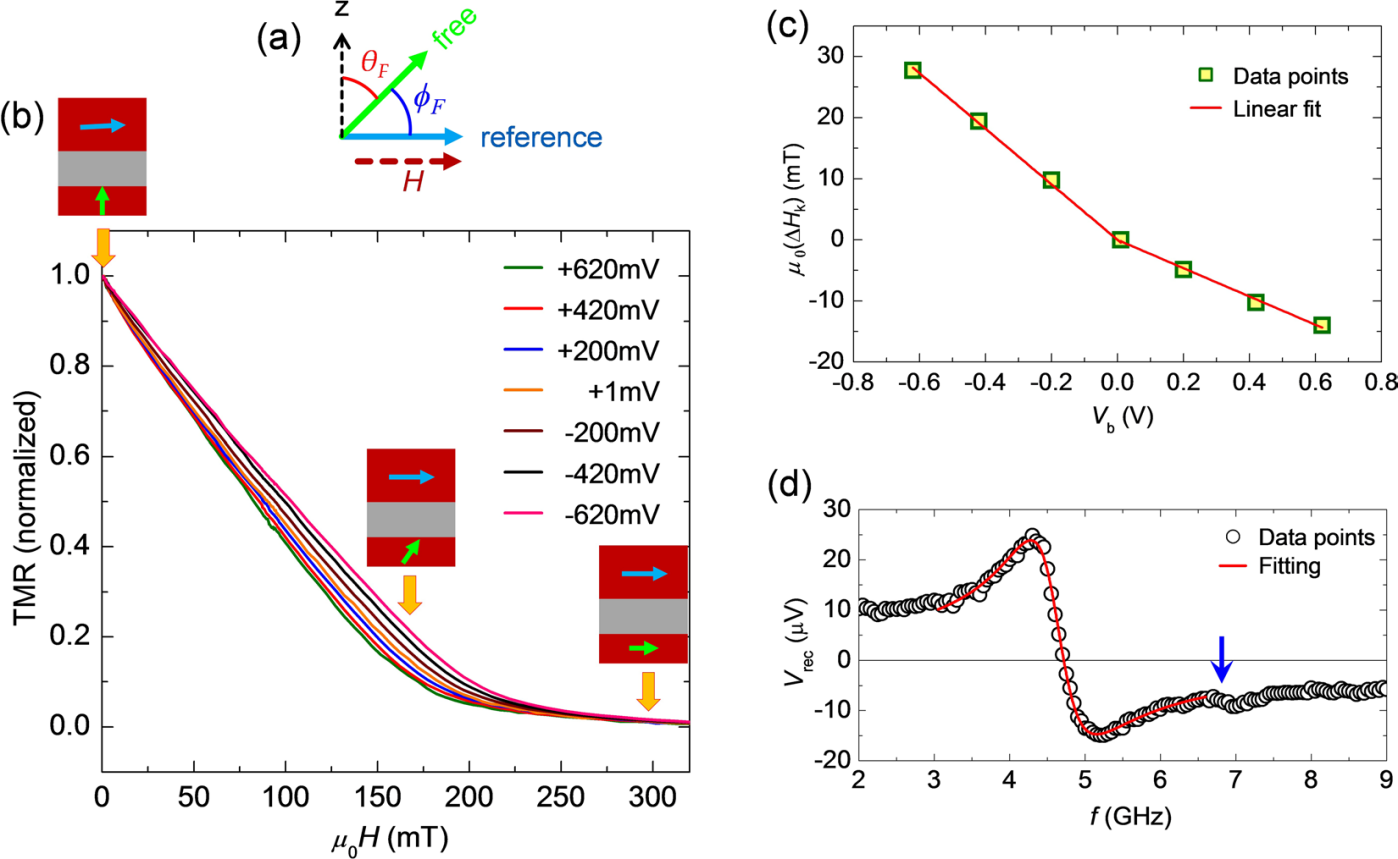
PMA versus voltage and typical rectified voltage signal in linear regime. (**a**) Schematic diagram for the geometry of free & reference layer magnetizations and bias magnetic field. (**b**) Normalized TMR as a function of *µ*_*0*_ *H* for different values of *V*_b_. The schematic diagrams show the alignment of magnetizations for free and reference layers. (**c**) Variation of change in *µ*_0_ *H*_k_ with *V*_b_. Solid lines correspond to the linear fitting to find out the slope. (**d**) Typical plot of *V*_rec_ as a function of *f* measured at *P*_rf_ = 5 µW. Solid line represents the fitted curve with Eq. 1.

To evaluate the variation of PMA field (*μ*_0_ *H*_k_) with voltage, we measured TMR of MTJ as a function of external magnetic field magnitude (*μ*_0_ *H*) from −320 mT to +320 mT along the *y*-axis (Fig. 1) under various dc bias voltages (*V*_b_) ranging from −0.62 V to +0.62 V. Note that positive voltage means the top electrode has positive potential with respect to the bottom electrode of MTJ. The resistance and area product (RA) for our device is about 100 kΩ.µm^2^. The TMR of a MTJ depends upon the relative angle between the magnetizations of two CoFeB layers^29^, *i.e*. ${\rm{TMR}}\propto \,\sin \,{\varphi }_{F}$, where *ϕ*_F_ is the angle between the magnetization﻿s of﻿ two CoFeB layers (Fig. 2(a)). Therefore the ratio of in-plane component of free layer magnetization (*M*_y_) and saturation magnetization (*M*_s_) can be evaluated from normalized TMR curve^30^ as ${M}_{y}\propto {M}_{s}\,\cos \,{\varphi }_{F}$. In Fig. 2(b), we plot experimentally measured normalized TMR as a function of +*μ*_0_ *H* for different values of *V*_b_. At *μ*_0_ *H* = 0 mT, all the curves show their maximum values which indicates that the magnetizations of both layers are aligned almost perpendicular to each other along their easy axes as schematically shown in the same figure. As *μ*_0_ *H* increases, the magnetization of free layer starts to incline along positive *y*-axis. Therefore, TMRs decrease monotonically with the increase of +*μ*_0_ *H* for all bias voltages until they reach to the minimum values at around +190 mT, indicating that the magnetizations of the both CoFeB layers are aligned along the +*y*-axis for *μ*_0_ *H* > + 190 mT. The difference in slopes among the curves originates from the modulation of *μ*_0_ *H*_k_ by *V*_b_ through VCMA effect^31^. We evaluate *μ*_0_ *H*_k_ for each value of *V*_b_ by using the method mentioned in refs 18, 30 and 31. The effective value of *μ*_0_ *H*_k_ at *V*_b_ = 0 V is about 185 mT. In Fig. 2(c), we plot *μ*_0_Δ*H*_k_, defined by the difference between *μ*_0_ *H*_k_ at certain bias voltage and zero bias voltage, as a function of *V*_b_. The magnitudes of the gradients of $|{\mu }_{0}{\rm{\Delta }}{H}_{k}|$ are 45 and 23 mT/V for negative and positive *V*_b_, respectively. The difference between the slopes of *μ*_0_Δ*H*_k_ for negative and positive *V*_b_ originates probably due to the two different interfaces (CoFeB/MgO & CoFeB/Ta) of free CoFeB layer in the MTJ^22, 31^.

### Resonance line-shape and line-width at lower excitation power

We measured *V*_rec_ as a function of *f* for different magnitudes of +*μ*_0_ *H*. Figure 2(d) shows a typical plot of *V*_rec_ as a function of *f* measured at *μ*_0_ *H* = 100 mT and rf power (*P*_rf_) = 5 µW. The rf power is low enough to excite FMR in the linear regime. The plot shows two resonance peaks. The resonance peak at 4.72 GHz with large peak-to-peak height (*V*_pp_) corresponds to FMR of free layer, whereas the resonance peak at 6.75 GHz with very small value of *V*_pp_ corresponds to FMR of reference layer. This shows that the reference layer is not efficiently excited by voltage due to near in-plane orientation of magnetization. Note that very low tunneling current density (~10^6^ A.m^−2^) can not excite reference layer by spin transfer torque (STT) or field like torque (FLT). The FMR spectrum of free CoFeB layer (Fig. 2(d)) can be fitted by linear combination of symmetric and anti-symmetric Lorentzian functions given by refs 18, 32, 33:

1 $${V}_{{\rm{rec}}}=\frac{{V}_{{\rm{s}}}}{1+{(f-{f}_{{\rm{FMR}}})}^{2}/{\sigma }^{2}}+\frac{{V}_{{\rm{as}}}(f-{f}_{{\rm{FMR}}})/\sigma }{1+{(f-{f}_{{\rm{FMR}}})}^{2}/{\sigma }^{2}},$$

where, *V*_s_ and *V*_as_ are the amplitudes of symmetric and anti-symmetric terms, *σ* is the half width at half maximum (HWHM) of the spectrum and *f*_FMR_ is the FMR frequency. For homodyne-detected signal, *V*_s_ originates from STT induced FMR, whereas *V*_as_ originates from the FMR induced by VCMA torque and/or FLT^18, 33, 34^. Peak-to-peak height (*V*_pp_) of *V*_rec_ curves can be obtained by summing up *V*_s_ and *V*_as_. Equation 1 fits well with the experimental resonance spectrum of free layer as shown by solid line in Fig. 2(d). According to the fitting result, *V*_as_ is around ten times larger than *V*_s_. Therefore, the contribution of STT is negligible or much smaller than the combined effect of VCMA torque and FLT. For further confirmation, we plot peak-to-peak value (*V*_pp_) of *V*_rec_ as a function of angle (*ϕ*_F_) made by free layer with film plane (see supplementary for details). The VCMA torque induced homodyne signal amplitude should be proportional to sin^2^ *ϕ*_F_ cos *ϕ*_F_ (refs 10 and 21), where *V*_pp_ should be maximum at *ϕ*_F_ ~ 55°. The maximum value of *V*_pp_ in our sample is obtained for *ϕ*_F_ ~ 59°, which implies voltage excitation of magnetization dynamics. The small deviation from theoretical value may occur due to the stray magnetic field from reference layer^10^.

Next, we discuss about the line-width (HWHM) of FMR spectra. The HWHM (*σ*) of the measured FMR spectra can be expressed as^35^

2 $$\sigma ={\sigma }_{ex}+{\sigma }_{in}={\sigma }_{ex}+\alpha {f}_{FMR}.$$

Here, *σ*_in_ is the intrinsic line-width, which originates from intrinsic Gilbert damping, *σ*_ex_ is the extrinsic line-width, which originates from various extrinsic contributions and *α* is the intrinsic Gilbert damping constant. The intrinsic line-width is proportional to the *f*_FMR_. According to the literature^24^, the intrinsic damping constant of 1.4 nm CoFeB is about 0.02, which gives the intrinsic resonance line-width (*σ*_in_) of about 100 MHz in absence of any extrinsic contribution. We extracted the value of *σ* as 425 MHz from the fitting of resonance spectrum with Eq. 1. This broad resonance line-width in our device indicates the presence of other extrinsic contributions, which may originate from inhomogeneous distribution of PMA and/or two-magnon scattering. In our case, the device was post-annealed at 300 °C in vacuum for 1 hour. Therefore, the PMA is expected to be uniform throughout the film. Two-magnon scattering which originates from the defects at the interfaces^36, 37^, may be present only if *M*_*s*_ is aligned at an angle (*ϕ*_F_) less than 45° with respect to film plane^37^. In our case, two-magnon scattering has negligible contribution as *ϕ*_F_ = 58° (>45°) (see supplementary informations). In this study, the magnetization dynamics are locally excited in the centre of a wider thin film, where spin waves are also excited outside the excitation area in addition to uniform FMR underneath the excitation area (see supplementary informations). The eigen-frequencies of the uniform FMR and spin wave modes are almost degenerated due to the small thickness of CoFeB film. This opens up a new channel for relaxation of spins correspond to uniform FMR via spin waves. Therefore, the resonance peak of uniform FMR mode is broadened. Probably, this broad peak of uniform FMR mode and other spin wave modes are not well resolved in our experiment. Therefore, we observed a single and broad resonance peak in our device instead of many isolated peaks.

### Resonance spectra as a function of excitation power

**Figure 3:**
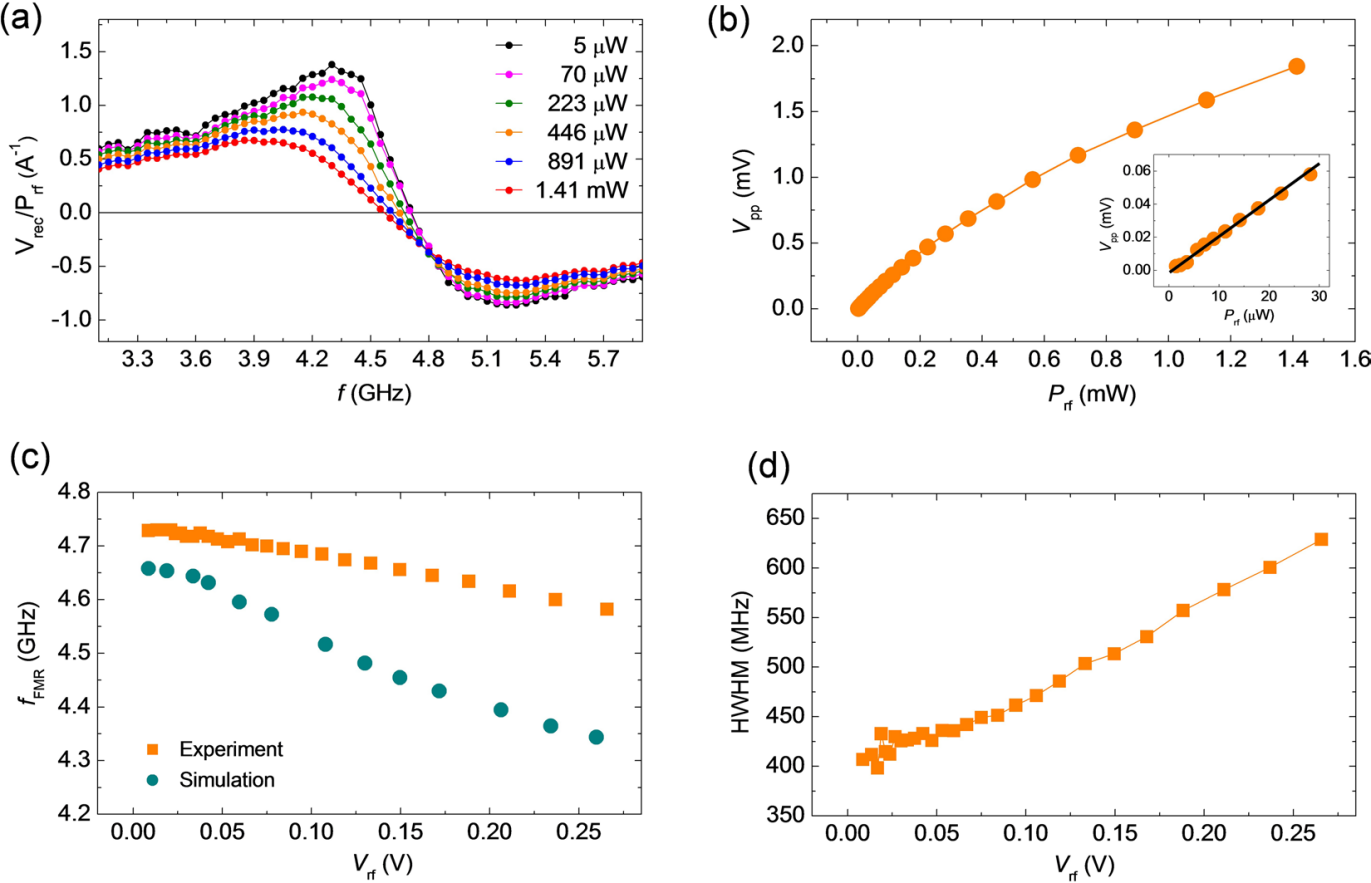
Experimental resonance spectra and dynamic parameters versus power. (**a**) Plot of experimentally measured *V*_rec_, rescaled by 1/*P*_rf_, versus *f* for different values of *P*_rf_. (**b**) Peak-to-peak value (*V*_pp_) of *V*_rec_ as a function of *P*_rf_. Inset shows the magnified graph of the same in lower *P*_rf_ regime. Solid line is a guide to eye for showing linear increment of *V*_pp_ with *P*_rf_. (**c**) Plot of experiment and simulation results show the variation of FMR frequency (*f*_FMR_) as a function of *V*_rf_. (**d**) Plot of HWHM as a function of *V*_rf_.

We measured resonance spectra for different values of *P*_rf_ ranging from 5 µW to 1.41 mW at *μ*_0_ *H* = 100 mT. In static condition, the free layer magnetization is oriented at an angle of 32° with respect to the film normal at this magnetic field value. We plot *V*_rec_, rescaled by a factor of 1/*P*_rf_, as a function of *f* for different values of *P*_rf_ in Fig. 3(a). With the increase of *P*_rf_, the resonance line-width is broadened maintaining anti-symmetric Lorentzian shape and the resonance frequency (*f*_FMR_) shifts towards the lower frequency. These features may be the fingerprints of nonlinear magnetization dynamics. However, we didn’t observe hysteresis between the resonance spectra for upward and downward sweeps of *f* (see supplementary information) in our MTJ unlike the previous reports^15, 20^. The reason behind this is probably thermal fluctuation of magnetizations due to Joule heating^15, 21^.

We fitted the resonance spectra with Eq. 1 to find out the parameters *V*_s_, *V*_as_, *f*_FMR_ and *σ*. Figure 3(b) shows the plot of peak-to-peak height (*V*_pp_) of *V*_rec_ curves as a function of *P*_rf_. The inset graph shows that the *V*_pp_ linearly increases with *P*_rf_ up to about *P*_rf_ = 30 μW. However, *V*_pp_ deviates from linear increment at higher values of *P*_rf_. In Fig. 3(c), we represent variation of *f*_FMR_ as a function of *V*_rf_, where *V*_rf_ = (P_rf_Z_0_)^1/2^ is the input rf voltage and *Z*_0_ (=50 Ω) is the characteristic impedance of waveguide. The graph shows that *f*_FMR_ remains almost constant (4.72 GHz) up to *V*_rf_ ≈ 0.04 V, (which correspond to 32 μW of rf power) and then monotonically (linearly) decreases with the increase of *V*_rf_. We also plot HWHM as a function of *V*_rf_ in Fig. 3(d). The HWHM remains almost constant (~425 MHz) up to *V*_rf_ ≈ 0.04 V. After that, HWHM increases almost linearly with the increase of *V*_rf_.

### Cone angle of precession

We also roughly estimated cone angle of precession in experiment from peak-to-peak (*V*_pp_) value of resonance spectra by using the following expression^10, 21^:

3 $$\sin \,{\theta }_{c}=\frac{2}{{r}_{TMR}\,\sin \,{\theta }_{F}}\frac{{R}_{AP}(R+{Z}_{0})}{{R}^{2}}\frac{{V}_{pp}}{{V}_{rf}}.$$

**Figure 4:**
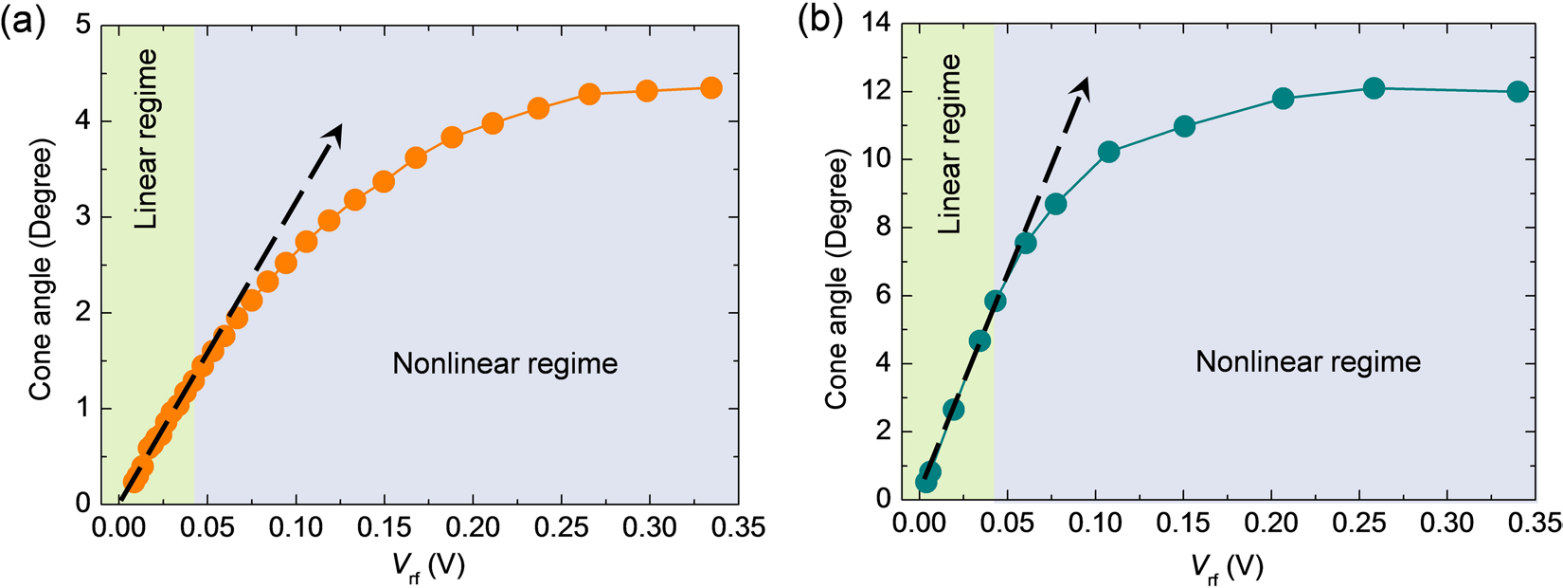
Cone angle of magnetization precession, evaluated from (**a**) experimental results and (**b**) micromagnetic simulations, are plotted as a function of applied rf voltage. Arrows with solid dotted lines represent the expected linear variation of cone angle as a function of *V*_rf_ in the linear regime. The linear and nonlinear regime of magnetization dynamics are demonstrated by two different background colour shades.

Here, *θ*_c_ is the cone angle of precession, *r*_TMR_ = (*R*_AP_−*R*_P_)/*R*_P_, *R*_P_ and *R*_AP_ are resistances for parallel and anti-parallel orientations of magnetization respectively, *R* is the resistance at *μ*_0_ *H* = 100 mT and *Z*_0_ is the characteristics impedance of waveguide. Figure 4(a) shows the plot of the *θ*_c_ as a function of *V*_rf_. Graph shows that *θ*_c_ linearly increases with *V*_rf_ up to *V*_rf_ = 0.04 V, which corresponds to *P*_rf_ ≈ 32 µW. Above 32 µW, *θ*_c_ deviates from linear increment with *V*_rf_ and becomes almost saturated above *V*_rf_ ≈ 0.27 V (*P*_rf_ ≈ 1.41 mW), where we get the maximum value of *θ*_c_ of about 4.35°. All these observations suggest that *P*_rf_ ≈ 32 µW (*V*_rf_ ≈ 0.04 V) is the threshold value of rf power above which the dynamics enters into nonlinear regime.

### Micromagnetic simulations

**Figure 5:**
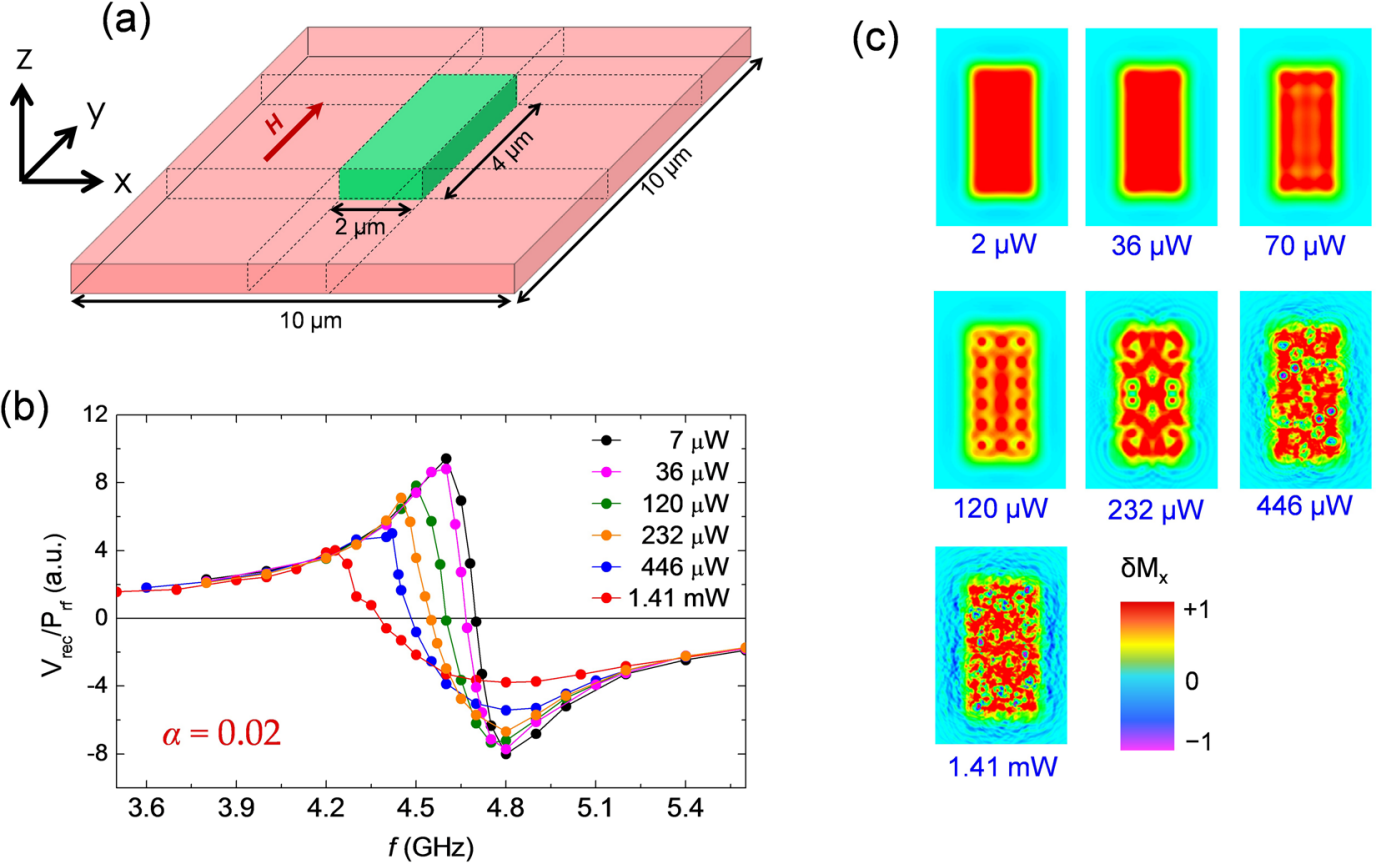
Micromagnetic simulation results. (**a**) Schematic diagram of the model sample for simulation. The dynamics were excited and the dynamic signals were extracted from the green area. Geometry of bias magnetic field is also shown. (**b**) Simulated *V*_rec_, rescaled by factor of 1/*P*_rf_, are plotted as a function of *f* for different values of *P*_rf_. (**c**) Spatial maps of the dynamic magnetization for different values of *P*_rf_. Displayed area of each map is 4 × 6 µm^2^ taken from the central part of 10 × 10 µm^2^ model sample. Dynamics were excited in the central 2 × 4 µm^2^ area of the maps.

To understand the underlying mechanism of the observed dynamic behaviours as a function of *P*_rf_, we performed micromagnetic simulations^38^ based on Landau-Lifshitz-Gilbert (LLG) equation (see methods). In the simulations, magnetization dynamics were excited by applying sinusoidal rf magnetic field (*h*_rf_ sin *2πft*) along *z*-axis with frequency *f* only in the central 2 × 4 µm^2^ area of CoFeB layer highlighted by green colour in Fig. 5(a). The magnitudes (*h*_rf_) of rf magnetic field were taken to be equivalent to the PMA field modulated by applied *V*_rf_ as calculated from experimental result presented in Fig. 2(c) and Eq. 4 given below^21^.

4 $${h}_{rf}={V}_{rf}(1+{\rm{\Gamma }})\frac{\partial {H}_{k}}{\partial {V}_{b}}\,\sin \,{\varphi }_{F}\,\cos \,{\varphi }_{F}.$$

Here, $\frac{\partial {H}_{k}}{\partial {V}_{b}}=34.2\mathrm{mT}/V$ is the average gradient of *μ*_0_Δ*H*_k_ for positive and negative *V*_b_, Γ = (*R* − *Z*_0_)/(*R* + *Z*_0_) is the reflection coefficient of *V*_rf_ from the MTJ. The experimentally measured rectified voltage (*V*_rec_) can be expressed as the time-averaged value of the product of oscillating TMR due to precession of magnetization and rf tunnel current as given below^18, 33^

5 $${V}_{{\rm{rec}}}=\langle R(t){I}_{rf}(t)\rangle =\langle \frac{R}{1+{p}^{2}\,\cos \,{\varphi }_{F}(t)}\frac{{V}_{{\rm{rf}}}}{R}\,\sin (2\pi ft)\rangle ,$$

where, *R* is the resistance, *p* is a spin polarization (we used 0.5), and *ϕ*_F_(*t*) is time-dependent relative angle between the magnetizations in the free and reference CoFeB layers. As the orientation of reference layer magnetization is nearly in-plane of the film, we simply adopt the angle (*ϕ*_F_) made by free layer with the film plane as equals to the angle made by free layer with the reference layer. *R*(*t*) = *R*/(1 + *p* ^2^ cos *θ*_F_(*t*)) represents the time-dependent resistance due to oscillation of *ϕ*_F_ and *I*_rf_(*t*) = *V*_rf_sin(2*πft*)/*R* represents the time-dependent tunnel current. Therefore, experimentally measured *V*_rec_ can be reproduced by micromagnetic simulations by using the time variation of *ϕ*_F_ when applying *V*_rf_. Note that *ϕ*_F_ is the angle made by average magnetization of the green area (Fig. 5(a)) with the film plane. We used *α* = 0.02 in the simulation.

Figure 5(b) shows the plot of simulated rectified voltage (*V*_rec_), rescaled by a factor of 1/*P*_rf_, as a function of excitation frequency (*f*) for different values of *P*_rf_. The simulated resonance spectra also look like anti-symmetric Lorentzian in the linear or lower power (up to 36 μW) regime. With the increase of *P*_rf_ the resonance spectra are broadened gradually like experimental results. The spectra also show decrease of *f*_FMR_ and peak-to-peak height of *V*_rec_/*P*_rf_ curves with *P*_rf_ similar to the experimental results. To get into the more details of the underlying mechanism, we show snapshots of the spatial distribution of dynamic magnetization for different values of *P*_rf_ in Fig. 5(c). The snapshots show that the dynamics is uniform and coherent up to 36 μW of rf power. Above 36 μW, the dynamics starts to become non-uniform and incoherent. The degree of incoherence gradually increases with further increase of *P*_rf_. The snapshots clearly show a smooth transition from coherent or linear magnetization dynamics to incoherent or nonlinear magnetization dynamics with a threshold power of about 36 μW. Therefore the dynamic dephasing due to the incoherent nonlinear dynamics reduces peak-to-peak height (*V*_pp_) of rectified voltage (*V*_rec_). Hence we observe a deviation of *V*_pp_ from linear increment with *P*_rf_ and deviation of *θ*_c_ from linear increment with *V*_rf_ above threshold value of *P*_rf_ in experiment as well as in simulation results. Note that this dynamic dephasing also broadens resonance line-width.

In Fig. 4(b) we also plot simulated value of *θ*_c_ as a function of *V*_rf_. The graph shows that *θ*_c_ linearly increases with *V*_rf_ up to *V*_rf_ = 0.04 V (*P*_rf_ ≈ 32 µW). Above the threshold power of 32 µW, *θ*_c_ deviates from linear increment with *V*_rf_ and becomes almost saturated above *V*_rf_ ≈ 0.25 V (*P*_rf_ ≈ 1.25 mW), where we get the maximum value of *θ*_c_ of about 12°. It shows that simulation result qualitatively reproduces the experimental result about the variation of *θ*_c_ with *V*_rf_ (Fig. 4(a)). However, simulation result shows much larger cone angle of precession as opposed to the experiment. This can be explained in the following way. We observed broad resonance line-width in experiment due to the spin wave generation outside the excitation area. In this way spin angular momentum corresponds to uniform FMR mode is transferred to its degenerate spin waves. Therefore in our experiment, the cone angle of precession corresponds to uniform FMR can not go beyond 4.35° even at very high excitation power. For exact reproduction of experimental result all the external parameters like extrinsic contributions of damping, temperature, stray field from reference layer and other boundary conditions have to be taken into account, which is not possible in OOMMF simulation. Therefore, broad resonance line-width due to extrinsic contributions like spin wave generation can not be reproduced in micromagnetic simulations. That is why the cone angle of precession in simulation can reach up to 12° unlike experiment.

We further plot simulated values of *f*_FMR_ as a function of *V*_rf_ in Fig. 3(c). The simulated results also show that *f*_FMR_ remains almost constant up to about *V*_rf_ = 0.04 V (threshold power). Above threshold power, *f*_FMR_ decreases almost linearly with *V*_rf_ like the experimental result due to the non-linear dynamics. However, we observed a steeper variation of *f*_FMR_ with *V*_rf_ in the simulation than that of the experiment. In the linear regime, the precessional axis of the magnetization makes an angle of 32° with respect to out-of-plane direction which is essentially the direction of the effective magnetic field (*H*_eff_) determined by bias magnetic field, PMA field and demagnetizing field. With the increase of *P*_rf_, the cone angle of precession increases which in fact decreases *M*_z_ (ref. 15). Therefore, *H*_eff_ and hence *f*_FMR_ decreases with the increase of *P*_rf_. In our simulation, the steeper decrease of *f*_FMR_ with *V*_rf_ was observed mainly due to the larger cone angle of precession as opposed to the experiment. A complete quantitative reproduction of experimental results is not possible as the effect of Joule heating and extrinsic contributions to the resonance line-width can not be taken into account. These Joule heating may also change the PMA of CoFeB film. Therefore the simulation result may differ from experimental results. Nevertheless, the micromagnetic simulations qualitatively reproduce all the important features of dynamics behaviour observed in the experiment.

## Discussions

A complete knowledge about the voltage induced nonlinear magnetization dynamics is essential for the application in future spintronics devices. However, the behaviour of nonlinear magnetization dynamics is quiet different than that of the linear dynamics. The magnetization dynamics in the nonlinear regime are governed by second order Suhl process^16, 39^ or four magnon scattering. In this process two *k* = 0 magnons are annihilated to create two *k* ≠ 0 (+*k* and −*k*) magnons^19, 40^. In other words a spontaneous transfer of angular momentum from coherent uniform precession of magnetizations to incoherent non-uniform precession of magnetizations occurs. This agrees with the observed behaviour in micromagnetic simulations. The dynamic dephasing among these incoherent *k* ≠ 0 magnons decreases the amplitude (*V*_pp_) or cone angle of precession (*θ*_c_) of dynamic magnetization than that of the expected values. The relaxation of magnetization dynamics in the nonlinear regime is also governed by intrinsic four magnon scattering^40^. The spin relaxation rate increases with the increase of four magnon scattering^39^. Again, the line-width of the resonance spectra is linearly proportional to the relaxation rate. As the four magnon scattering process increases with the increase of *P*_rf_, we observed a monotonic increment of HWHM as a function of *V*_rf_ or *P*_rf_.

In conclusion, we have shown a systematic transition from linear magnetization dynamics to nonlinear magnetization dynamics for local excitation of magnetization by experiment and micromagnetic simulation study. Simulations results demonstrate that the coherent and linear magnetization dynamics becomes incoherent and nonlinear above a certain threshold value of rf power. This incoherent nonlinear dynamics significantly reduces dynamic signal i.e. cone angle of magnetization precession and increases resonance line-width. Voltage induced local magnetization dynamics is essential for exciting spin waves in future spintronics devices. We show that locally excited uniform magnetization dynamics also excites spin waves, almost degenerate to uniform FMR mode, outside the excitation area in ultrathin magnetic films. The resonance line-width correspond to uniform FMR mode is enhanced due to the channelling of angular momentum from uniform FMR inside excitation area to spin wave mode outside the excitation area. We have further shown that this broad resonance line-width does not allow cone angle of magnetization precession to exceed only above few degrees in our device. We think our study will be very useful for the development of all-voltage-controlled spin waves based logic devices.

## Methods

### Sample fabrication

The devices were fabricated by a multistep fabrication method. At first, film-stacking structure used in this study were prepared on a thermally oxidized Si(001) substrate by rf sputtering at room temperature at a base pressure of 10^−9^ Torr. The structure consists of the following layers (nominal thicknesses in nanometres are stated in parentheses): Si/Ta(5)/Ru(20)/Ta(5)/Co_20_Fe_60_B_20_(1.4)/MgO(2)/Co_20_Fe_60_B_20_(3)/Ta(5)/Ru(5). In second step, the free layers with dimension 50 × 100 μm^2^ were defined by maskless photolithography followed by Ar^+^ ion milling down to Si-substrate. In the third step, reference layers with dimension 2 × 4 μm^2^ were defined in the middle of free layers (Fig. 1(a)) by maskless photolithography followed by Ar^+^ ion milling down to MgO layer and subsequent deposition of 40 nm thick Al_2_O_3_. The dimension of reference layer defines the dimension of MTJ. In the fourth step, 100 nm thick Al_2_O_3_ was deposited everywhere except on top of the reference layer (for top electrode) and in the vicinity of the edge of free layer (for bottom electrode). Finally, the contacts were designed by photolithography followed by deposition of Ti(5)/Au(100) by electron beam evaporation. Fabricated devices were post-annealed at 300 °C in vacuum under a perpendicular magnetic field of 600 mT for one hour. High temperature annealing over 300 °C decreased µ_0_ *H*_k_ due to intermixing of the interface between CoFeB and Ta^41^. For a good thermal stability of the surface magnetic anisotropy, one can use W buffer layer instead of Ta^42^.

### Experimental Measurement

The ferromagnetic resonance in CoFeB layers were excited by applying rf voltage across the MTJ. The rf voltage (*V*_rf_) produces rf electric field (*E*_rf_) at the interfaces of the CoFeB/MgO junction. The *E*_rf_ modulates interfacial PMA of CoFeB layer. When frequency of *V*_rf_ matches the FMR frequency (*f*_FMR_) of free layer, magnetization dynamics is excited. Magnetization dynamics produces an oscillatory TMR (depends upon the relative angle between the magnetizations of two layers) at the same frequency than that of the *V*_rf_. The mixing of oscillatory TMR and small rf tunnel current generates finite dc voltage (called as rectified voltage), which is measured by a multimeter connected to the dc port of the bias tee. To remove the background not coming from FMR, we measured reference spectra at *μ*_0_ *H* = 300 mT correspond to each rf power (*P*_rf_) and subtracted from spectrum corresponds to each bias magnetic field and *P*_rf_. At *μ*_0_ *H* = 300 mT, the magnetizations of free layer and reference layer are expected to be parallel to each other. Therefore precession of free layer magnetization does not produce any time-dependent TMR.

### Micromagnetic Simulations

The micromagnetic simulations were performed by using Object Oriented Micromagnetic Framework (OOMMF) software based on Landau-Lifshitz-Gilbert equation of motion^38^ given by:

$$\frac{{\rm{d}}{\bf{M}}}{{\rm{d}}t}=-\,\gamma {\bf{M}}\times {{\bf{H}}}_{{\rm{eff}}}+\alpha {\bf{M}}\times \frac{{\rm{d}}{\bf{M}}}{{\rm{d}}t}\cdot $$

The STT term is neglected because of its small contribution in our MTJ. In this equation, **M** is saturation magnetization, *γ* is the gyromagnetic ratio, *α* is the Gilbert damping constant and **H**_**eff**_ is the effective magnetic field composed of bias magnetic field, PMA field and demagnetizing field. For the simulations, a model sample with dimension 10 µm (*x*) × 10 µm (*y*) × 1.4 nm (*z*) was considered (Fig. 5(a)). The mess size was taken as 20 nm × 20 nm × 1.4 nm. We adopted the anisotropy energy density of 1.012 × 10^5 ^J/m^3^ in out-of-plane direction, saturation magnetization *µ*_0_ *M*_s_ of 1.5 T, gyromagnetic ratio of 28 GHz.T^−1^ and exchange stiffness constant of 20 pJ.m^−1^. The exchange stiffness constant was found from ref. 43, whereas other parameters were extracted from experimental results (see supplementary information). At first, the ground state of magnetization was prepared by applying a bias magnetic field along *y*-direction. The magnetization dynamics was then excited by applying a sinusoidal rf magnetic field (*h*_z_ = *h*_rf_ sin 2*πft*) (Eq. 4) with frequency *f*. As *V*_rf_ modulates PMA of free CoFeB layer only in the area underneath the reference CoFeB layer, *h*_z_ was applied only in the central 2 × 4 µm^2^ area of CoFeB layer marked by green colour in Fig. 5(a). The magnitudes of *h*_z_ *i.e. h*_rf_ were chosen equivalent to the PMA field modulated by applied *V*_rf_ as calculated from experimental result shown in Fig. 2(b) and Eq. 4. The time varying average magnetization components were also extracted from the green area to mimic the experimental condition.

## Electronic supplementary material

Effect of excitation power on voltage induced local magnetization dynamics in an ultrathin CoFeB film
