# Supplementary material for: Effect of excitation power on voltage induced local magnetization dynamics in an ultrathin CoFeB film
Source: Sci Rep. 2017 May 24;7:2318. doi: 10.1038/s41598-017-02427-3 (PMC5443791; doi:10.1038/s41598-017-02427-3)
Supplement: Supplementary file 1 — Effect of excitation power on voltage induced local magnetization dynamics in an ultrathin CoFeB film [file 41598_2017_2427_MOESM1_ESM.pdf]

Supplementary informations

Effect of excitation power on voltage induced local magnetization dynamics in an ultrathin CoFeB film

Bivas Rana[1], Yasuhiro Fukuma[1,2], Katsuya Miura[3], Hiromasa Takahashi[3] and YoshiChika Otani[1,4]

[1]Center for Emergent Matter Science, RIKEN, 2-1 Hirosawa, Wako 351-0198, Japan;

[2]Frontier Research Academy for Young Researchers, Kyushu Institute of Technology, 680-4 Kawazu, Iizuka 820-8502, Japan;

[3]Research and Development Group, Hitachi, Ltd., 1-280 Higashi-koigakubo, Kokubunji-shi, Tokyo 185-8601, Japan;

[4]Institute for Solid State Physics, University of Tokyo, Kashiwa 277-858, Japan;

1. Confirmation of voltage excitation:

To confirm that the magnetization dynamics is predominantly excited by voltage, we measured rectified voltage (V_{rec}) as a function of excitation frequency (f) for different magnitudes of applied magnetic field ($\mu_0 H$) along y-axis. The curves were measured at 5 μW of rf power (P_{rf}). Figure S1(a) shows the plot of measured V_{rec} curves. In the figure, each curve of V_{rec} is artificially shifted by 7 μV vertically. The curves show two resonance peaks. The peaks marked by shaded area correspond to the ferromagnetic resonance (FMR) of free CoFeB layer, whereas the peaks marked by down arrows correspond to the FMR of reference CoFeB layer. We fit all the resonance curves with Eq. 1 (in the main manuscript) to find out the amplitudes of anti-symmetric (V_{as}) & symmetric (V_s) Lorentzian terms, line-width (σ) and resonance frequency (f_{FMR}) of free CoFeB layer. In Fig. S1(b), we plot f_{FMR}

of free CoFeB layer as a function of $\mu_0 H$. The data points can be fitted with Kittel's formula by taking into account PMA. The formula is given by[1]

$$f_{FMR}^2 = \left(\frac{\mu_0 \gamma}{2\pi}\right)^2 \left[(H_k - M_s)^2 - (H + H_{stray})^2\right] \qquad \text{(S1)}$$

where, γ is gyromagnetic ratio, H_k is perpendicular magnetic anisotropy (PMA) field, M_s is saturation magnetization given by $1.5/\mu_0$ T and H_{stray} is the stray magnetic field from reference layer. The fitted curve is shown in the same figure by a solid line. The extracted parameters from fitting are $\gamma = 28$ GHz.T^{-1}, $\mu_0 H_k = 1.686$ T and $\mu_0 H_{stray} = 1.2$ mT. This gives the effective value of PMA, $\mu_0 (H_k)_{eff} = \mu_0 (H_k - M_s)$, as 186 mT. This means the magnetizations of two CoFeB layers are aligned along in-plane direction for $\mu_0 H \geq 186$ mT. It can also be confirmed from dc TMR measurement as a function of $\mu_0 H$ as shown in Fig. 2b. The angle (ϕ_F) made by free layer magnetization with in-plane direction can be expressed as

$$\phi_F = \sin^{-1}\left(\frac{H + H_{stray}}{H_k - M_s}\right) \qquad \text{(S2).}$$

We calculate ϕ_F for all values of $\mu_0 H$ by using Eq. S2 and plot peak-to-peak value (V_{pp}) of V_{rec} as a function of ϕ_F in Fig. S1(d). The torque induced by voltage-controlled magnetic anisotropy (VCMA) on free layer magnetization is proportional to $\sin\phi_F \cos\phi_F$, whereas the detection sensitivity of MTJ is proportional to $\sin\phi_F$. Therefore V_{pp} should be proportional to $\sin^2\phi_F \cos\phi_F$ i.e. the maximum value of should be observed at $\phi_F = 55^\circ$ as reported earlier[2,3]. We fit our data points (V_{pp}) with $A\sin^2(\phi_F - \beta)\cos(\phi_F - \beta)$ function, where A is arbitrary constant and β is the offset angle. The fitted curve is shown by a solid line in the same figure. We get maximum value of V_{pp} at $\phi_F = 59^\circ$ with an offset angle $\beta = 4^\circ$. A small offset may be present due to the stray field from reference layer and a small contribution of spin transfer torque (STT)[2]. This result confirms that the magnetization dynamics are excited predominantly by voltage or electric field in our device.

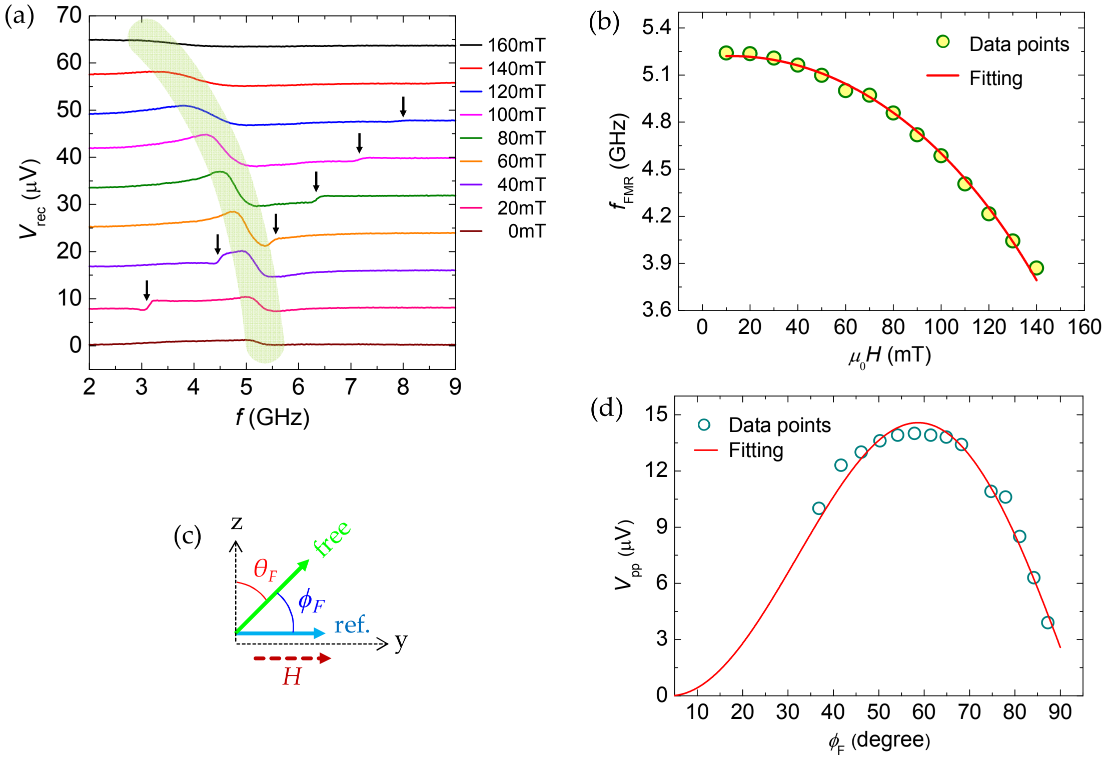

Figure S1. Confirmation of voltage excitation. (a) Rectified voltage as a function of frequency (f) for different values of bias magnetic field ($\mu_0 H$). **(b)** Ferromagnetic resonance frequencies (f_{FMR}) as a function of $\mu_0 H$ are plotted. Solid line shows fitting with the Eq. S1. **(c)** The geometry of the magnetizations of free and reference CoFeB layers and direction of H are schematically represented. **(d)** Peak-to-peak values (V_{pp}) of V_{rec} are plotted as a function of angle (ϕ_F) made by free layer magnetization with in-plane direction. Solid line correspond to the fitting with function $A\sin^2(\phi_F - \beta)\cos(\phi_F - \beta)$.

2. Hysteresis behaviour:

One of the fingerprints of non-linear magnetization dynamics is observation of hysteresis between the FMR spectra measured with upward (low to high) and downward (high to low) sweeps of f without lock-in-amplifier. In Fig. S2 we show the experimentally measured FMR spectra for upward and downward sweep of f at P_{rf} = 1.41 mW. We do not observe any hysteresis between these two spectra. The reason behind this may be thermal effect on the magnetization dynamics[3,4] due to Joule heating as reported earlier also.

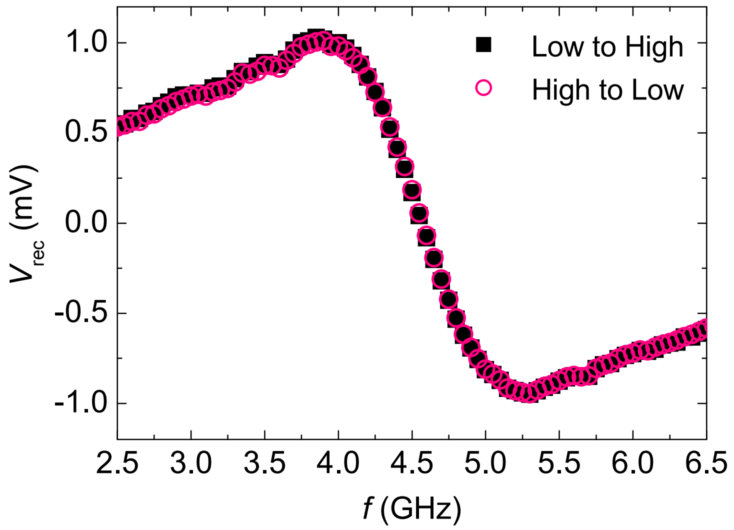

Figure S2. Investigation of hysteresis behaviour. Experimentally measured V_{rec} as a function of f for upward (low to high) and downward (high to low) sweeps of f.

3. Presence of spin wave modes:

We performed micromagnetic simulations to investigate whether spin wave modes are excited or not. For the simulations we chose a model device with dimensions same as described in the 'method' section in main manuscript. The magnetization dynamics were excited by applying a Gaussian pulse magnetic field with peak amplitude of 0.5 mT and HWHM of 25 ps. The resonance frequencies were extracted from the fast Fourier transform (FFT) of the simulated time varying magnetization dynamics. In the first case, we extracted (detected) the time varying magnetization dynamics from the central area (2×4 μm^2) where the dynamics were exited (Fig. S3(a)). The FFT of the magnetization dynamics (Fig. S3(c)) shows a single resonance peak correspond to uniform FMR mode at $f = 4.65$ GHz. In the second case, we extracted (detected) time varying magnetization dynamics from the 2×4 μm^2 area just outside the excitation area as schematically shown in Fig. S3(b). The FFT of the corresponding magnetization dynamics, as shown in Fig. S3(d), demonstrates that a number of spin wave modes with frequencies higher than that of the uniform mode frequency are excited. The frequencies of those spin wave modes are very close to the frequency of uniform mode. Therefore, in real experiment the line-width correspond to uniform FMR mode is broadened due to the transfer of angular momentum from uniform mode to spin wave modes.

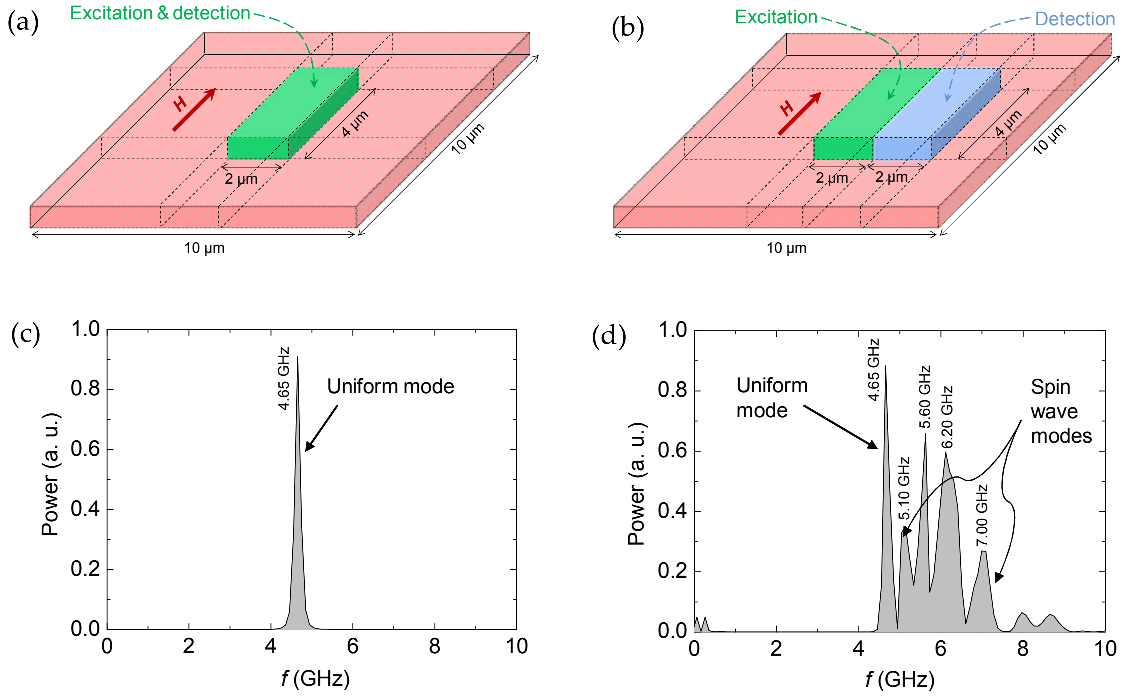

Figure S3. Micromagnetic simulation results on presence of spin wave modes. (a,b) Schematic diagrams of the model sample for simulating the presence of spin wave modes outside excitation area. In the first case (Fig. a), we excited and extracted (detected) dynamic signal from central area (2 × 4 μm^2). In the second case (Fig. b), we excited in the central area and extracted (detected) the dynamics signal from outside the excitation area. **(c)** The fast Fourier Transform (FFT) of dynamic magnetization signal detected from central area (2 × 4 μm^2) of excitation. **(d)** The fast Fourier Transform (FFT) of dynamic magnetization signal detected from outside of the central area (2 × 4 μm^2) of excitation.

Supplementary References:

1. Stancil, D. D. & Prabhakar, A. *Spin waves: theory and applications* (Springer, New York, 2008).
2. Nozaki, T. *et al.* Electric-field-induced ferromagnetic resonance excitation in an ultrathin ferromagnetic metal layer. *Nat. Phys.* **8**, 491-496 (2012).
3. Hirayama, E. *et al.* Electric-field induced nonlinear ferromagnetic resonance in a CoFeB/MgO magnetic tunnel junction. *Appl. Phys. Lett.* **107**, 132404 (2015).
4. Chen, W., de Loubens, G., Beaujour, J.-M. L., Sun, J. Z. & Kent, A. D. Spin-torque driven ferromagnetic resonance in a nonlinear regime. *Appl. Phys. Lett.* **95**, 172513 (2009).
